# Investigations on Arthropods Associated with Decay Stages of Buried Animals in Italy

**DOI:** 10.3390/insects12040311

**Published:** 2021-04-01

**Authors:** Teresa Bonacci, Federica Mendicino, Domenico Bonelli, Francesco Carlomagno, Giuseppe Curia, Chiara Scapoli, Marco Pezzi

**Affiliations:** 1 Department of Biology, Ecology and Earth Sciences, University of Calabria, Via P. Bucci, 87036 Arcavacata di Rende, Cosenza, Italy; teresa.bonacci@unical.it (T.B.); federica.mendicino@gmail.com (F.M.); domenico.bonelli@unical.it (D.B.); francescocarlomagno@gmail.com (F.C.); 2 Azienda Sanitaria Provinciale di Cosenza, Servizio Veterinario, Via J. F. Kennedy, Traversa II, 87036 Roges di Rende, Cosenza, Italy; peppinocuria@libero.it; 3 Department of Life Sciences and Biotechnology, University of Ferrara, Via L. Borsari 46, 44121 Ferrara, Italy; chiara.scapoli@unife.it

**Keywords:** arthropods, burial, decay, forensic entomology, insects, pig

## Abstract

**Simple Summary:**

The burial of corpses may interfere with the succession of sarcosaprophagous fauna and forensic evaluation of post-mortem interval. For the first time in Italy, an experimental study was conducted on arthropods associated with buried pig carcasses in a rural area near Cosenza (Southern Italy). One carcass was left above the ground and five were buried: one of the buried ones was periodically exhumed to evaluate the effects of disturbance on decay processes and on arthropod fauna, and the other four were exhumed only once at given time intervals. The results revealed differences in taxa and colonization of arthropod fauna in the above ground and periodically exhumed carcasses. No arthropod colonization was detected in the carcasses exhumed only once, showing that a burial at about 25 cm depth could be sufficient to prevent colonization by sarcosaprophagous taxa.

**Abstract:**

Burial could be used by criminals to conceal the bodies of victims, interfering with the succession of sarcosaprophagous fauna and with the evaluation of post-mortem interval. In Italy, no experimental investigation on arthropods associated with buried remains has been conducted to date. A first experimental study on arthropods associated with buried carcasses was carried out in a rural area of Arcavacata di Rende (Cosenza), Southern Italy, from November 2017 to May 2018. Six pig carcasses (*Sus* *scrofa* Linnaeus) were used, five of which were buried in 60-cm deep pits, leaving about 25-cm of soil above each carcass, and one was left above ground. One of the buried carcasses was periodically exhumed to evaluate the effects of disturbance on decay processes and on arthropod fauna. The other four carcasses were exhumed only once, respectively after 43, 82, 133, and 171 days. As expected, the decay rate was different among carcasses. Differences in taxa and colonization of arthropod fauna were also detected in the above ground and periodically exhumed carcasses. In carcasses exhumed only once, no arthropod colonization was detected. The results showed that a burial at about 25 cm depth could be sufficient to prevent colonization by sarcosaprophagous taxa and these data could be relevant in forensic cases involving buried corpses.

## 1. Introduction

Diptera and Coleoptera are the most studied insect orders in forensic entomology because they are associated to dead bodies for both trophic and reproductive activities [1,2,3] Insects are well known to colonize a corpse immediately after death [4] although intrinsic and extrinsic factors may affect their arrival and activities on the remains [5]. Temperature, humidity, and environmental conditions around the dead body affect the decay rate and the species composition [6,7]. Criminals sometimes use burial to conceal the bodies of their victims: this practice favors a reduction of the decay rate [8] and often prevents the arrival of sarcosaprophagous fauna on the body. When burial prevents access of insects to a body, entomological methods to estimate post-mortem interval (PMI) are not sufficiently reliable [9,10]. When colonization occurs, investigators may use the discovered specimens for forensic analyses, but insect relative abundance and diversity are often different between buried and exposed corpses [11]. Previous studies on buried corpses and flesh pieces reported the presence of many necrophagous species, mostly belonging to families of Diptera, such as Phoridae, Muscidae, and Sarcophagidae [11,12,13,14,15,16,17,18,19,20,21,22,23].

Forensic entomology practitioners may also have trouble in correctly evaluating PMI in the case of buried corpses because of lack of reference data (as occurring in Italy) and interference of local factors. These challenges may be met by a correct integration of field casework and laboratory studies, also requiring experimental studies on animal models in scenarios relevant for forensic entomology. These experimental studies may provide useful data for future applications to forensic investigations.

In order to identify arthropod taxa and succession on buried remains, we carried out for the first time in Italy an investigation using an animal model, namely pig carcasses buried in a rural area in Calabria (Southern Italy).

## 2. Materials and Methods

### 2.1. Study Site

**Figure 1:**
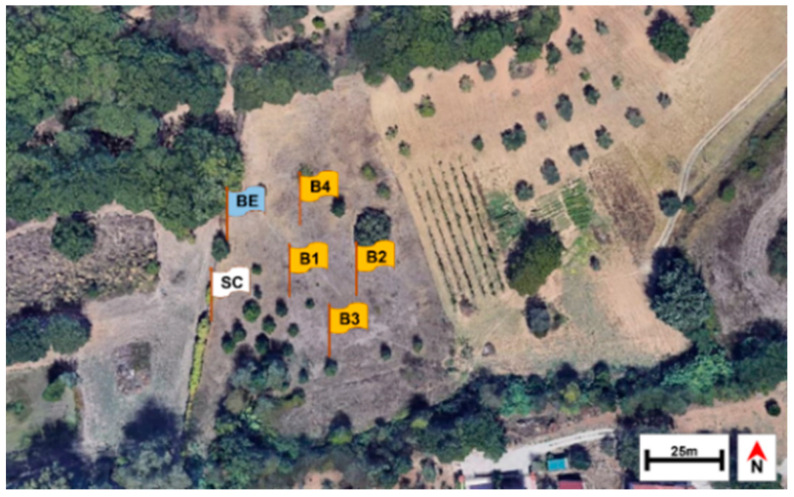
Satellite view (Google Earth) of the experimental site showing the rural area where the pig carcasses were positioned (Flags). SC, position of carcass set above the soil; B1–B4, positions of buried carcasses exhumed only once; BE, position of periodically exhumed carcass.

**Figure 2:**
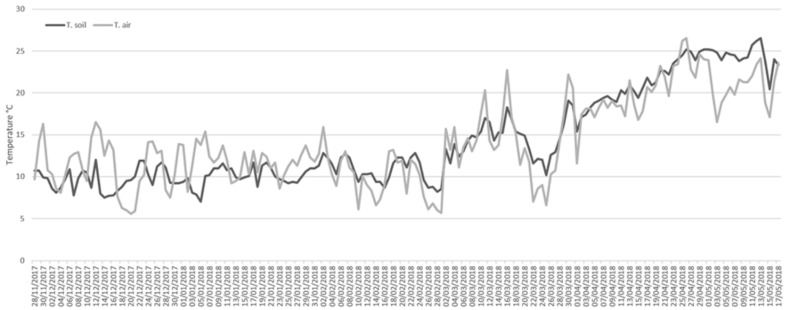
Data of soil (T. soil) and air temperature (T. air) and recorded at the experimental site from 28 November 2017, to 17 May 2018. Data are expressed as average of daily measurements at each hour from 9 a.m. to 5 p.m. Soil temperature data were measured with a manual probe every day, each hour from 9 a.m. to 5 p.m., at 10-cm depth above carcass B4. Air temperature data were provided by Centro Funzionale Multirischi—ARPACAL (Catanzaro, Italy), station Cosenza 118.

**Figure 3:**
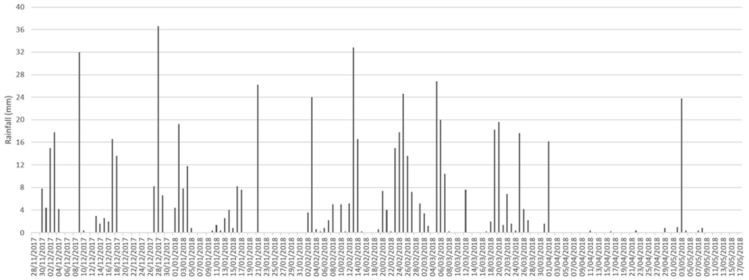
Daily rainfall data (mm) provided by ARPACAL pluviometer station (website http://www.arpacal.it/; accessed on 7 August 2020), referring to station Cosenza 118 (cod. 1017).

The investigation was carried out from 28 November 2017, to 17 May 2018 in a rural area in Southern Italy, located within the campus of the University of Calabria (Arcavacata di Rende, Cosenza, 39°21′35.31″ N and 16°13′53.48″ W). The site was about 220 m a.s.l. and consisted of a grassy area with juvenile trees of *Quercus pubescens* Wild. (Fagales: Fagaceae), *Olea europaea* L. (Scrophulariales: Oleaceae) and *Populus alba* L. (Salicales: Salicaceae) (Figure 1). The soil granulometry, analyzed by the Laboratory of Geodynamics and Earth Surface Processes of the Department of Ecology, Biology and Earth Sciences of the University of Calabria, revealed a composition of 54% sand, 23% silt, 14% clay, and 9% gravel. The soil was therefore identified as “sandy clay loam”, according to the classification by the United States Department of Agriculture. Based on previous studies [21], six 120-kg female pigs, *Sus scrofa* Linnaeus (Artiodactyla: Suidae), were used. The experimental animals were purchased at a farm adjacent to the sampling site and the animals were sacrificed at the local slaughterhouse under veterinary control, according to Regulation (EU) 2017/625 of the European Parliament on animal health and welfare (Document 32017R0625), published in Official Controls Regulation (EU) L95 (ISSN 1977-0707) on 7 April 2017. Five pits, each 1.2 × 1.5 m large and 60-cm deep were mechanically excavated two days before the beginning of the experiment. The pits were spaced at least 15 m from each other on all sides (Figure 1) to prevent interference in the succession fauna. All animals were set inside the pits on the left side over a 25-mm mesh chicken wire. Since the pig carcasses laid on the left side were about 35-cm high at their maximum, the thickness of soil above each carcass was about 25 cm. Four carcasses were respectively labelled as B1, B2, B3, and B4 (Figure 1). For comparison, a fifth carcass, labelled SC, was positioned at the same time and location on the soil surface and left to open air, protected by the chicken wire which was removable for inspections. A sixth carcass, labelled BE, was placed in another pit in similar conditions to those of B1, B2, B3, and B4, but was periodically exhumed to evaluate the effects of disturbance on decay processes and on the associated arthropod fauna (Figure 1). Carcasses B1, B2, B3, and B4 were exhumed only once during the experimental period, respectively after 43, 82, 133, and 171 days. Carcass BE was exhumed every 10 or 15 days according to weather conditions. Data of air temperature for each day during the experimental period were provided by Centro Funzionale Multirischi—ARPACAL (Agenzia Regionale per la Protezione dell’Ambiente della Calabria) (Catanzaro, Italy), referring to the station Cosenza 118. Figure 2 shows the average values of air temperature between 9 a.m. and 5 p.m. for each day of the experimental period. Soil temperature was measured with a digital thermometer with a probe (IHM Moineau Instruments, Boutonne, France) every day, during the experimental period, each hour from 9 a.m. to 5 p.m., at 10-cm depth. The average of data of soil temperature are shown in Figure 2. Daily data about mm of rainfall were provided by ARPACAL pluviometer station (website http://www.arpacal.it/; accessed on 7 August 2020), referring to station Cosenza 118 (cod. 1017) (Figure 3).

### 2.2. Arthropod Sampling and Carcass Decay

Every day, carcass SC and the surfaces of all pits were examined for arthropod presence twice a day (from 9 a.m. to 1 p.m., and from 3 p.m. to 5 p.m.). The decay process of carcasses SC and BE was also examined according to a previous protocol [21]. For each carcass, the decomposition rate and the duration of each decomposition stage were recorded. Sampling of arthropods for carcass BE was carried out every 10–15 days: the carcass was exhumed and carefully observed only for 20 min, to avoid any interference on arthropod colonization. Adults were captured by an entomological net and tweezers and larvae were hand collected by tweezers during the observation time.

The same sampling procedure of arthropod fauna was carried out at the exhumations of carcasses B1, B2, B3, and B4. At each exhumation of BE and of the other buried carcasses, soil samples from the sides, above and (when possible) under the carcass, in positions corresponding to the head, anus, dorsum, back and abdomen (for a total of about 10 kg of soil) were collected and examined by a Berlese funnel in the Applied and Forensic Entomology Laboratory of the University of Calabria [24].

The adult arthropods collected during the observation time and by the Berlese funnel on soil samples were stored in 60% ethanol and taxonomically identified at the maximum possible level using keys [25,26,27,28,29,30,31,32,33,34,35,36,37,38,39,40,41]. Concerning the dipteran larvae collected by tweezers, they were reared to adults in plastic boxes containing a layer of sand covered with about 100 g bovine liver, at a temperature similar to that of field collection. Once the larvae reached the pupal stage, the liver residuals were removed, leaving only the sand where the larvae reached the pupal stage. The emerged Diptera adults were stored in 60% ethanol and identified at the maximum possible level using keys [35,38]. Concerning the larvae of Dermestidae, those collected by tweezers were reared to adults in plastic boxes containing a layer of sand covered with about 100 g dry pork skin, at a temperature similar to that of field collection. Once the larvae reached the pupal stage, the skin residuals were removed, leaving only the sand where the larvae reached the pupal stage. The emerged Dermestidae adults were stored in 60% ethanol and identified at the maximum possible level using keys [41]. Concerning the arthropods collected by the Berlese funnel, that causes the death of arthropods, preserving them in a liquid, the collected larvae were identified using taxonomical keys for larvae at the maximum possible level [42,43].

## 3. Results

### 3.1. Decay Rate

All along the experimental period, for air temperature the daily average ranged from a minimum of 5.6 to a maximum of 26.5 °C, and for soil temperature the daily average ranged from a minimum of 7.0 to a maximum of 26.5 °C (Figure 2). Rainfall throughout the duration of the study was frequent and abundant (Figure 3), with a total of 600.6 mm, according to rain data provided by ARPACAL pluviometer station Cosenza 118 (cod. 1017). 

The decay rate of the buried carcasses compared to carcass SC was much slower. The depth of pit, the soil compactness (due to abundant rainfall) and the average low temperature recorded at 25-cm depth may have affected the decomposition process. 

The decay of carcass BE lasted 171 days, while that of carcass SC required 82 days. Four decomposition stages were observed for carcass SC (fresh, bloated, decay/advanced decay and dry), and for BE (fresh, bloated, adipocere/advanced decay and dry). 

Carcasses B1 and B2, respectively exhumed after 43 and 82 days, were observed at the adipocere stage, while B3 and B4, respectively exhumed after 133 and 171 days, were observed at the advanced decay stage.

### 3.2. Insect Succession

From carcass SC, a total of 6162 arthropod specimens were collected and identified as belonging to five orders and 25 families (Table 1, Table 2 and Table 3). The most abundant order was that of Diptera, with 12 families (Table 1).

During the fresh stage (about 18 h from positioning) the taxa of adults collected were *Calliphora vicina* Robineau-Desvoidy, *Calliphora vomitoria* (Linnaeus), *Lucilia sericata* (Meigen) (Diptera: Calliphoridae), *Hydrotaea dentipes* (Fabricius), *Musca domestica* Linnaeus (Diptera: Muscidae), *Homoneura* sp. van der Wulp (Diptera: Lauxaniidae), Heleomyzidae (Diptera) and *Carpelimus* sp. Leach (Coleoptera: Staphylinidae).

The first oviposition by Diptera was observed as early as five minutes after the positioning of carcass SC on the soil, inside an eye. 

The first instar of dipteran larvae was observed 96 h after egg laying, due to the low temperatures on the day of positioning and on the following days. After 96 h, the carcass SC was in the early bloated stage.

During the bloated stage (lasting about 13 days), the taxa of adults were *C. vomitoria, C. vicina*, *Chrysomya albiceps* Wiedemann, *Lucilia caesar* (Linnaeus), *L. sericata* (Diptera: Calliphoridae), *Fannia canicularis* (Linnaeus) (Diptera: Fanniidae), *H. dentipes*, *M. domestica*, *Muscina prolapsa* (Harris), *Phaonia subventa* (Harris), *Polietes meridionalis* Peris & Llorente, *Thricops* sp. Róndani (Diptera: Muscidae), *Piophila casei* (Linnaeus), *Stearibia nigriceps* (Meigen) and, *Prochyliza* sp. Walker (Diptera: Piophilidae), and *Sepsis duplicata* Haliday (Diptera: Sepsiidae). During this stage, adults belonging to the families Dryomizidae, Sarcophagidae (Diptera) and Braconidae (Hymenoptera) and to the genus *Carpelimus* sp. were also identified.

During this stage, the larvae detected belonged to the species *C. vicina*, *C. vomitoria* and *L. sericata* (Table 1).

In the decay/advanced decay stage (lasting about 38 days) the most abundant taxa of adults collected on carcass SC were *C. vomitoria*, *C. vicina*, *Ch. albiceps*, *F. canicularis*, *H. dentipes*, *L. sericata*, *L. caesar*, *M. domestica*, *Pi. casei*, and *S. nigriceps*. 

Adults belonging to the family Braconidae and adults of *Nitidula flavomaculata* Rossi (Coleoptera: Nitidulidae) were also collected. Among larvae, the most abundant taxa were *C. vomitoria* and *H. dentipes*.

In the dry stage (lasting about 27 days), the collected taxa of adults were *C. vicina*, *C. vomitoria*, *H. dentipes* and Simulidae for Diptera, and *Creophilus maxillosus* (Linnaeus), *Paederus* sp. Fabricius (Staphylinidae) and *N. flaveomaculata* for Coleoptera. The only larvae collected belonged to *C. vicina*, *C. vomitoria*, and *H. dentipes*.

Based on the immature stages collected, the only species breeding on carcass SC were respectively *C. vomitoria*, *C. vicina*, *H. dentipes,* and *L. sericata*.

Concerning the other carcasses exhumed only once (B1, B2, B3, and B4, respectively exhumed at 43, 82, 133, and 171 days after the date of positioning), carcass B1, exhumed 43 days after the positioning, was in the decay/adipocere stage. Carcass B2, exhumed 82 days after the positioning, was also in the decay/adipocere stage. Carcass B3, exhumed 133 days after the positioning, was in the advanced decay stage, as well as carcass B4, exhumed 171 days after the positioning. 

After a careful examination of all carcasses, no signs of colonization by arthropods was detected on them at the date of exhumation. No arthropods were also detected or collected during the daily inspections of the surface of all pits of B1–B4.

Concerning the carcass BE, which was exhumed every 10–15 days to evaluate the effects of disturbance on decay processes and associated arthropod fauna, during daily inspections on the pit surface a total of nine taxa of adults were collected: *C. vomitoria*, *C. vicina*, *Helina* sp. Robineau-Desvoidy, *Hydrotaea meteorica* (Linnaeus), *M. domestica*, *P. subventa* (Diptera: Muscidae), *Sarcophaga tibialis* Macquart (Diptera: Sarcophagidae), *Messor* sp. Forel (Hymenoptera: Formicidae), and *Scolopendra cingulata* Latreille (Scolopendromorpha: Scolopendridae). 

Carcass BE appeared in the bloated stage from 10 to 65 days from positioning (respectively corresponding to the first and sixth exhumation), thus this stage lasted about 56 days. In this time interval, during the third exhumation (43 days after positioning), carcass BE was mostly colonized by larvae of *C. vicina* and of the coleopteran families Staphilinidae and Carabidae. 

The same carcass appeared in the adipocere/advanced decay stage from 74 to 163 days after positioning (respectively corresponding from the seventh to the twelfth exhumation), thus this stage lasted about 90 days. In this time interval, the most frequent orders were Diptera and Coleoptera (Table 1 and Table 2), and the order with the highest number of taxa detected at the larval stage was Diptera. 

The taxa recorded at the larval stage were *C. vomitoria*, *L. sericata*, *F. canicularis* and *Fannia lineata* (Stein) (Diptera: Fanniidae), *Hydrotaea aenescens* (Wiedemann), *H. capensis*, *H. ignava*, *Mu. prolapsa*, and *Muscina stabulans* (Fallén) (Diptera: Muscidae). 

Concerning Coleoptera, the only species found at the larval stage was *Dermestes frischi* (Kugelann) (Coleoptera: Dermestidae). Larvae belonging to the infraorder Tipulomorpha (Diptera) were also detected. At the dry stage, occurring 171 days after positioning (corresponding to the thirteenth exhumation) no larval stage was detected. Concerning adults, for Diptera, only individuals of *Megaselia scalaris* (Loew) (Phoridae) and other individuals belonging to the family Sciaridae were found. 

Some Coleoptera of the families Histeridae, Dermestidae and Staphylinidae were also found. From dipteran larvae collected from carcasses and reared in laboratory the parasitoid *Nasonia vitripennis* (Walker) (Hymenoptera: Pteromalidae) emerged and this species was also detected in soil samples analyzed by the Berlese funnel.

## 4. Discussion

The size and instar of dipteran larvae and/or patterns of postmortem insect succession are relevant to estimate the interval of arthropod colonization on a corpse [2]. Although biotic and abiotic factors may affect both decay and insect successional pattern, the entomological method used to estimate PMI could be applied when corpses colonized by insects are discovered in both outdoor and indoor environments. Burial is a common method used by criminals to conceal a corpse, but burial depth and soil hardness are physical barriers that significantly affect temperature and insect succession [13]. In our study, in which pig carcasses weighing about 120 kg each were buried at a depth of 60 cm, a layer of about 25 cm was on the top of each carcass exhumed only once (B1–B4). According to our data, this layer of soil was sufficient to prevent the arrival of arthropods and slow down the decay rate of carcasses.

The decay rate was therefore different among the carcass left above ground (SC), the carcass periodically exhumed (BE) and those exhumed only once (B1–B4). No arthropods were observed on the surface of the pits of carcasses exhumed only once, probably because the soil compactness at 25-cm depth prevented the spread of odors arising from decaying remains. It is also possible that anoxic conditions may have developed though high saturation of the soil by water associated to low evaporation. This hypothesis is supported by the low temperatures and frequent rains detected during the experimental period and by the observed adipocere stages.

Concerning the periodically exhumed carcass, the effects of the disturbance on the pit surface were the reduced soil compactness and the mixing of the surface with the deeper soil in contact with the carcass, thus spreading odors, attracting arthropods and increasing the number of colonizing taxa.

Although the above ground and buried carcasses were set simultaneously in their positions, a different insect successional pattern was found on the exposed carcass in comparison to the buried ones. Dipteran species such as *C. vicina*, *C. vomitoria* and *L. sericata* arrived and bred on both carcasses SC and BE. On carcass SC, *Ch. albiceps* arrived but did not breed, and *H. dentipes* was the only one of its genus breeding on it. Contrary to what occurred in the carcasses exhumed only once (B1–B4), in carcass BE the larvae, whose presence signaled breeding activity, belonged to the species *F. canicularis*, *F. lineata*, *H. aenescens*, *H. capensis*, *H. ignava*, *Mu. prolapsa* and *Mu. stabulans*. Some colonization by Tipulomorpha was also found. The highest number of larvae detected belonged to *H. capensis*. The presence of *H. ignava* and other unidentified species of the genus *Hydrotaea* was previously reported in buried remains at 30-cm and 60-cm depth [21]. Colonization by *Mu. prolapsa* and *Mu. stabulans* on buried animal remains was also reported [44]. The relatively low insect diversity on carcass SC in comparison to carcass BE is interesting and could be related to the different decay rate, but this aspect requires more in-depth studies. It is interesting to notice that in carcasses exhumed only once (B1-B4) no colonization by arthropods was detected, not even that due to common forensic indicator species reported in buried remains, such as *Conicera tibialis* Schmitz (Diptera: Phoridae) and *Me. scalaris* [2,16,21,23,45]. However, these two dipteran species were found at the adult stage in carcasses SC and BE: *Me. scalaris* was found in both carcasses, but *Co. tibialis* only on BE. The absence of these two species is intriguing: probably the soil texture or some other factor may be involved, and this point requires further investigation. In Italy, *Me. scalaris* was reported as the only species colonizing a human corpse buried in a wooden coffin at 30–40 cm depth [46]. This species, also reported in pig carcasses buried at 60-cm depth [21], has medical and forensic interest because it is known to cause myiasis [47]. The species *Co. tibialis*, commonly known as “coffin fly”, is usually detected in exhumed human corpses [16,20,48]. In our investigation, this was observed visiting carcass BE but no larvae were detected. Because the decay of buried carcasses usually occurs at a much slower rate than air exposed ones [13], dipteran larvae normally associated with the earlier stages of decomposition were detected later on carcass BE in comparison to carcass SC. It is also interesting to notice that species considered “thermophilic”, such as *Ch. albiceps*, *L. caesar* and *L. sericata* [49] were found in carcasses SC and BE in winter months. Moreover, according to a previous study conducted in Region Calabria, *C. vomitoria* is a species abundant in *Fagus* sp. L. (Fagales: Fagaceae) woodlands and infrequent in rural and urban areas [50]. However, in our study this species resulted abundant both as adults and larvae, although the investigation was carried out in a rural area near urban settlements.

Concerning Coleoptera, in carcass BE a higher number of taxa of the family Staphylinidae was found in comparison to the other carcasses. The species of this family colonizing carcasses are characterized by predatory habits towards adults and immature stages of sarcosaprophagous insects [1,2]. A species that was found on both carcasses SC and BE is *Cr. maxillosus*, commonly reported as a carrion colonizer [51,52,53]. Its role as a forensic bioindicator of PMI is under evaluation [54]. In Italy, *Cr. maxillosus* was previously reported on two human bodies found in rural areas in Region Veneto [55] and on another mummified body found in a woodland area of Northwestern Italy [56].

A possible explanation of the high number of taxa of Staphylinidae on carcass BE, periodically exhumed and reburied, was the fact that the soil was mixed, becoming more humid, less compact and carrying attractive odors, thus more suitable for colonization by hypogean Coleoptera. Moreover, on carcass BE the necrophagous coleopteran *D. frischi* and the coleopteran species with necrophilus habits *Margarinotus brunneus* (Fabricius), *Margarinotus ventralis* (Marseul)*, Saprinus semistriatus* (Scriba) (Histeridae), *Necrobia ruficollis* (Fabricius) and *Necrobia violacea* Linnaeus (Cleridae) were found. Among Coleoptera, the only species found breeding on BE was *D. frischi*. Adults and preimaginal stages of this species were previously detected in Northwestern Italy on the mummified human body found outdoor in woodlands [56]. In the region of Calabria, preimaginal stages of *D. frischi* were previously reported in another mummified human body, found outdoors in a rural area [57].

The hypothesis that periodical exhumation and reburial of carcass BE has a role in making soil more suitable to colonization by predator Coleoptera may be supported by the presence of some adults belonging the family Carabidae, such as *Abax* sp. Bonelli, *Acinopus* sp. Dejean, *Ophonus* sp. Dejean, *Ophonus cribricollis* (Dejean) and *Trechus quadristriatus* (Schrank), and an adult of the water beetle, *Sphaeridium lunatum* Fabricius (Coleoptera: Hydrophilidae), usually found in very humid environments [58].

Concerning the effects of the soil granulometry on arthropod colonization, in a previous study on a similar soil type a large colonization was detected on pig carcasses buried at depths of 30 and 60 cm [21]. However, in our study no arthropod colonization was detected in carcasses B1–B4, exhumed only once, probably because the soil became too compact due to winter rains and low temperatures. On the contrary, on carcass BE the arthropod colonization was present because of soil reworking during periodical exhumation and reburial.

Overall, the results show that in our experimental conditions a burial at about 25-cm depth is sufficient to prevent colonization by sarcosaprophagous taxa. These findings, obtained for the first time in Italy on pig carcasses, have forensic relevance because they provide useful data when the depth of burial, the type of soil and the weather conditions interfere with a correct evaluation of PMI by entomological methods in cases involving buried corpses. In our experimental conditions, in buried carcasses the delay in the decay process was affected by the lack of necrophagous insects, in turn affected by the compactness of the soil. A whole range of factors should be considered when entomological evidence is used for forensic purposes. The rate of decomposition and the regular sequence of sarcosaprophagous fauna related to decomposition stages is affected by many factors, in turn related to the location of the corpse and/or its burial [59].

Our results represent an experimental investigation of the effects of burial on arthropod colonization of corpses, which should be extended to different environments, such as urban, suburban, and forest ones, and to different seasons, in order to support future forensic investigations.

## Figures and Tables

**Table 1:** Taxa of Diptera found as adults (A) and larvae (L) in carcasses SC and BE, with number of individuals in brackets, at each of the four decay stages. The decay/advanced decay stage of carcass SC corresponds to the adipocere/advanced decay stage of carcass BE. Specimens were collected by an entomological net and/or tweezers, or by a Berlese funnel (*) or by mixed methods (**). SC, carcass set above the soil; BE, periodically exhumed carcass.

Infraorder/ Family	Genus/ Species	Stage of Decomposition							
		Fresh		Bloated		Decay/Advanced Decay		Dry	
		SC	BE	SC	BE	SC	BE	SC	BE
Calliphoridae	*Calliphora vicina*	A(3)	--	A(129); L(6)	L(92)	A(38); L(1)	--	L(4)	--
	*Calliphora vomitoria*	A(16)	--	A(258); L(14)	--	A(93); L(534)	L(617 **)	L(3695)	--
	*Chrysomya albiceps*	*--*	--	A(74)	--	A(17)	--	--	--
	*Lucilia caesar*	A(7)	--	A(103)	--	A(10)	--	--	--
	*Lucilia sericata*	*--*	--	A(107); L(4)	--	A(18)	L(7)	--	--
Fanniidae	*Fannia canicularis*	*--*	*--*	A(26)	*--*	A(24)	L(26)	*--*	*--*
	*Fannia lineata*	*--*	*--*	--	*--*	A(1)	L(5)	*--*	*--*
Muscidae	*Hydrotaea aenescens*	*--*	*--*	*--*	*--*	--	L(123)	*--*	*--*
	*Hydrotaea capensis*	*--*	*--*	*--*	*--*	A(1)	L(1373)	*--*	*--*
	*Hydrotaea dentipes*	A(1)	--	A(149)	--	A(147); L(50)	--	L(87)	--
	*Hydrotaea ignava*	*--*	*--*	*--*	*--*	A(1)	L(19)	*--*	*--*
	*Musca domestica*	A(10)	--	A(235)	--	A(45)	--	--	--
	*Muscina levida*	*--*	*--*	A(1)	*--*	A(1)	--	*--*	*--*
	*Muscina prolapsa*	*--*	*--*	A(6)	*--*	--	L(26)	*--*	*--*
	*Muscina stabulans*	*--*	--	A(1)	--	--	L(118)	--	--
	*Phaonia subventa*	*--*	*--*	A(7)	*--*	A(4)	*--*	*--*	*--*
	*Polietes meridionalis*	*--*	--	A(5)	--	--	--	--	--
	*Azelia* sp.	*--*	*--*	--	*--*	A(1)	*--*	*--*	*--*
	*Helina* sp.	*--*	*--*	A(3)	*--*	--	*--*	*--*	*--*
	*Thricops* sp.	*--*	*--*	A(10)	*--*	--	*--*	*--*	*--*
Phoridae	*Megaselia scalaris*	*--*	*--*	A(3)	A(1)	--	A(3)	--	A(1)
	*Conicera tibialis*	*--*	*--*	--	--	--	A(3 **)	--	--
	*Chaetopleurophora* sp.	*--*	*--*	A(2)	*--*	*--*	*--*	*--*	*--*
Piophilidae	*Piophila casei*	*--*	*--*	A(7)	*--*	A(6)	--	*--*	*--*
	*Stearibia nigriceps*	*--*	*--*	A(46)	*--*	A(10)	--	*--*	*--*
	*Prochyliza* sp.	*--*	*--*	A(3)	*--*	--	*--*	*--*	*--*
Sepsidae	*Sepsis duplicata*	*--*	*--*	A(11)	*--*	A(3)	*--*	*--*	*--*
	*Sepsis cynipsea*	*--*	*--*	A(2)	*--*	--	*--*	*--*	*--*
Anthomyiidae	*Anthomyia* sp.	*--*	*--*	A(2)	*--*	A(1)	*--*	*--*	*--*
Sarcophagidae	*Sarcophaga* sp.	*--*	*--*	A(1)	*--*	--	*--*	*--*	*--*
Cecidomyidae	*--*	*--*	*--*	*--*	--	--	A(2)	--	--
Drosophilidae	*--*	*--*	*--*	*--*	A(1)	--	A(4)	--	--
Dryomyzidae	--	*--*	*--*	A(4)	*--*	*--*	*--*	*--*	*--*
Ephydridae	*--*	*--*	*--*	*--*	A(2 *)	--	--	--	--
Heleomyzidae	--	A(1)	--	--	--	--	--	--	--
Lauxaniidae	--	A(1)	--	--	--	--	--	--	--
Psychodidae	*--*	*--*	*--*	*--*	A(1)	--	A(12 **)	--	--
Scatopsidae	*--*	*--*	*--*	*--*	A(2)	--	A(2)	--	--
Sciaridae	*--*	*--*	*--*	*--*	A(7 *)	--	A(30 **)	--	A(13 *)
Simulidae	--	*--*	*--*	A(2 *)	--	A(8 *)	A(1)	A(1 *)	--
Sphaeroceridae	*--*	*--*	*--*	*--*	A(1 *)	--	A(36 *)	--	--
Tipulomorpha	*--*	*--*	*--*	*--*	--	--	L(13 *)	--	--

**Table 2:** Taxa of Coleoptera found as adults (A) and larvae (L) in carcasses SC and BE, with number of individuals in brackets, at each of the four decay stages. The decay/advanced decay stage of carcass SC corresponds to the adipocere/advanced decay stage of carcass BE. Specimens were collected by an entomological net and/or tweezers, or by a Berlese funnel (*) or by mixed methods (**). SC, carcass set above the soil, BE, periodically exhumed carcass.

Family	Genus/ Species	Stage of Decomposition							
		Fresh		Bloated		Decay/Advanced Decay		Dry	
		SC	BE	SC	BE	SC	BE	SC	BE
Cleridae	*Necrobia ruficollis*	*--*	*--*	*--*	*--*	*--*	A(2)	--	--
	*Necrobia violacea*	*--*	*--*	*--*	*--*	*--*	A(4)	--	--
Dermestidae	*Dermestes frischi*	*--*	--	--	--	--	A(9); L(10)	--	A(2)
Geotrupidae	*Anoplotrupes stercorosus*	*--*	*--*	A(1)	--	*--*	*--*	*--*	*--*
Histeridae	*Margarinotus brunneus*	*--*	*--*	*--*	*--*	*--*	A(6)	--	A(3)
	*Margarinotus ventralis*	*--*	*--*	*--*	*--*	*--*	A(1)	--	A(1)
	*Saprinus semistriatus*	*--*	*--*	*--*	*--*	*--*	A(10)	--	A(2)
Hydrophilidae	*Sphaeridium lunatum*	*--*	*--*	*--*	*--*	*--*	A(1)	--	--
Nitidulidae	*Nitidula flavomaculata*	*--*	*--*	*--*	--	A(11)	--	A(19)	--
Silphidae	*Necrodes littoralis*	*--*	*--*	*--*	--	A(1)	--	--	--
	*Thanatophilus rugosus*	*--*	*--*	*--*	--	--	A(1)	--	--
Staphylinidae	*Creophilus maxillosus*	*--*	*--*	*--*	--	A(14)	A(5)	A(3)	--
	*Anotylus* sp.	*--*	*--*	*--*	A(3 **)	--	A(3)	--	--
	*Carpelimus* sp.	A(1)	--	A(10)	--	--	--	--	--
	*Heterothops* sp.	*--*	*--*	*--*	--	--	A(4)	--	--
	*Paederus* sp.	*--*	*--*	*--*	--	A(1)	--	A(2)	--
	*Platystethus* sp.	*--*	*--*	*--*	--	--	A(9)	--	--
	*Philonthus* sp.	*--*	*--*	*--*	A(1 *)	--	A(1)	--	--
	*Quedius* sp.	*--*	*--*	*--*	A(1)	--	A(4)	--	A(1)
	Aleocharinae	*--*	*--*	*--*	A(16)	--	A(10)	--	--
	Piestinae	*--*	*--*	*--*	--	--	A(1)	--	--
Carabidae	*Abax* sp.	*--*	*--*	*--*	A(1)	*--*	*--*	*--*	*--*
	*Acinopus* sp.	*--*	*--*	*--*	A(1)	*--*	*--*	*--*	*--*
	*Ophonus cribricollis*	*--*	*--*	*--*	A(1)	*--*	*--*	*--*	*--*
	*Ophonus* sp.	*--*	*--*	*--*	A(1)	*--*	*--*	*--*	*--*
	*Trechus quadristriatus*	*--*	*--*	*--*	A(1)	*--*	*--*	*--*	*--*
Buprestidae	--	*--*	*--*	A(1)	*--*	*--*	*--*	*--*	*--*
Chrysomelidae	--	*--*	*--*	A(1)	*--*	*--*	*--*	*--*	*--*
Curculionidae	--	*--*	*--*	A(1)	*--*	A(1)	*--*	*--*	*--*
Scarabeidae	--	*--*	*--*	A(1)	--	--	A(3)	--	--

**Table 3:** Other arthropod taxa found as adults (A) in carcasses SC and BE, with number of individuals in brackets, at each of the four decay stages. The decay/advanced decay stage of carcass SC corresponds to the adipocere/advanced decay stage of carcass BE. Specimens were collected by an entomological net and/or tweezers, or by a Berlese funnel (*) or by mixed methods (**). SC, carcass set above the soil; BE periodically exhumed carcass.

Order	Family	Genus/ Species	Stage of Decomposition							
			Fresh		Bloated		Decay/Advanced Decay		Dry	
			SC	BE	SC	BE	SC	BE	SC	BE
Araneae	Amaurobiidae	--	--	--	--	--	A(2)	--	--	--
	Lycosidae	--	--	--	--	A(7)	--	--	--	--
	Thomisidae	--	*--*	*--*	--	--	A(1)	--	--	--
	Salticidae	--	--	--	--	--	A(1)	--	--	--
Dermaptera	Forficulidae	*Forficula auricularia*	*--*	*--*	--	A(1)	--	--	--	--
Hymenoptera	Braconidae	--	*--*	*--*	A(18)	A(1 *)	A(8)	A(1)	--	--
	Formicidae	*Messor* sp.	*--*	*--*	--	A(1)	A(1)	--	--	A(1)
		*Camponotus* sp.	*--*	*--*	--	A(2)	--	--	--	--
		*Pheidole* sp.	*--*	*--*	--	--	--	A(36)	--	--
	Pteromalidae	*Nasonia* *vitripennis*	*--*	*--*	--	--	--	A(19 **)	--	A(2)
Rhynchota	Cicadellidae	*Eupteryx* sp.	*--*	*--*	A(1)	--	*--*	*--*	*--*	*--*
	Delphacidae	--	*--*	*--*	A(1)	--	*--*	*--*	*--*	*--*
Geophilomorpha	--	--	*--*	*--*	--	A(1)	*--*	*--*	*--*	*--*

## Data Availability

All data presented in this study are available in the article.
